# Digital Phenotyping of Mental and Physical Conditions: Remote Monitoring of Patients Through RADAR-Base Platform

**DOI:** 10.2196/51259

**Published:** 2024-10-23

**Authors:** Zulqarnain Rashid, Amos A Folarin, Yuezhou Zhang, Yatharth Ranjan, Pauline Conde, Heet Sankesara, Shaoxiong Sun, Callum Stewart, Petroula Laiou, Richard J B Dobson

**Affiliations:** 1 Institute of Psychiatry, Psychology and Neuroscience, King's College London, De Crespigny Park, Denmark Hill, London, SE5 8AF, United Kingdom, 44 02078480924; 2 Institute of Health Informatics, University College London, London, United Kingdom; 3 NIHR Biomedical Research Center at South London and Maudsley NHS Foundation Trust and King's College London, London, United Kingdom; 4 Health Data Research UK London, University College London, London, United Kingdom; 5 NIHR Biomedical Research Center at University College London Hospitals, NHS Foundation Trust, London, United Kingdom; 6 Department of Computer Science, University of Sheffield, Sheffield, United Kingdom

**Keywords:** digital biomarkers, mHealth, mobile apps, Internet of Things, remote data collection, wearables, real-time monitoring, platform, biomarkers, wearable, smartphone, data collection, open-source platform, RADAR-base, phenotyping, mobile phone, IoT

## Abstract

**Background:**

The use of digital biomarkers through remote patient monitoring offers valuable and timely insights into a patient’s condition, including aspects such as disease progression and treatment response. This serves as a complementary resource to traditional health care settings leveraging mobile technology to improve scale and lower latency, cost, and burden.

**Objective:**

Smartphones with embedded and connected sensors have immense potential for improving health care through various apps and mobile health (mHealth) platforms. This capability could enable the development of reliable digital biomarkers from long-term longitudinal data collected remotely from patients.

**Methods:**

We built an open-source platform, RADAR-base, to support large-scale data collection in remote monitoring studies. RADAR-base is a modern remote data collection platform built around Confluent’s Apache Kafka to support scalability, extensibility, security, privacy, and quality of data. It provides support for study design and setup and active (eg, patient-reported outcome measures) and passive (eg, phone sensors, wearable devices, and Internet of Things) remote data collection capabilities with feature generation (eg, behavioral, environmental, and physiological markers). The back end enables secure data transmission and scalable solutions for data storage, management, and data access.

**Results:**

The platform has been used to successfully collect longitudinal data for various cohorts in a number of disease areas including multiple sclerosis, depression, epilepsy, attention-deficit/hyperactivity disorder, Alzheimer disease, autism, and lung diseases. Digital biomarkers developed through collected data are providing useful insights into different diseases.

**Conclusions:**

RADAR-base offers a contemporary, open-source solution driven by the community for remotely monitoring, collecting data, and digitally characterizing both physical and mental health conditions. Clinicians have the ability to enhance their insight through the use of digital biomarkers, enabling improved prevention, personalization, and early intervention in the context of disease management.

## Introduction

### Background

Digital biomarkers offer a host of advantages for measuring our health over traditional assessment approaches that are typically confined to clinical settings, including decentralization, scalability, sampling frequency and real-time measurement, and affordability. However, significant challenges remain with implementing digital biomarkers.

Digital biomarkers developed from sensor data can help with prevention and early intervention to better diagnose and manage disease. Collected data should be reliable and of high quality reflecting the true condition of the patients, and many studies have attempted to measure the effectiveness of digital biomarkers for various clinical use cases [1].

### RADAR-Base Platform

**Figure 1.**
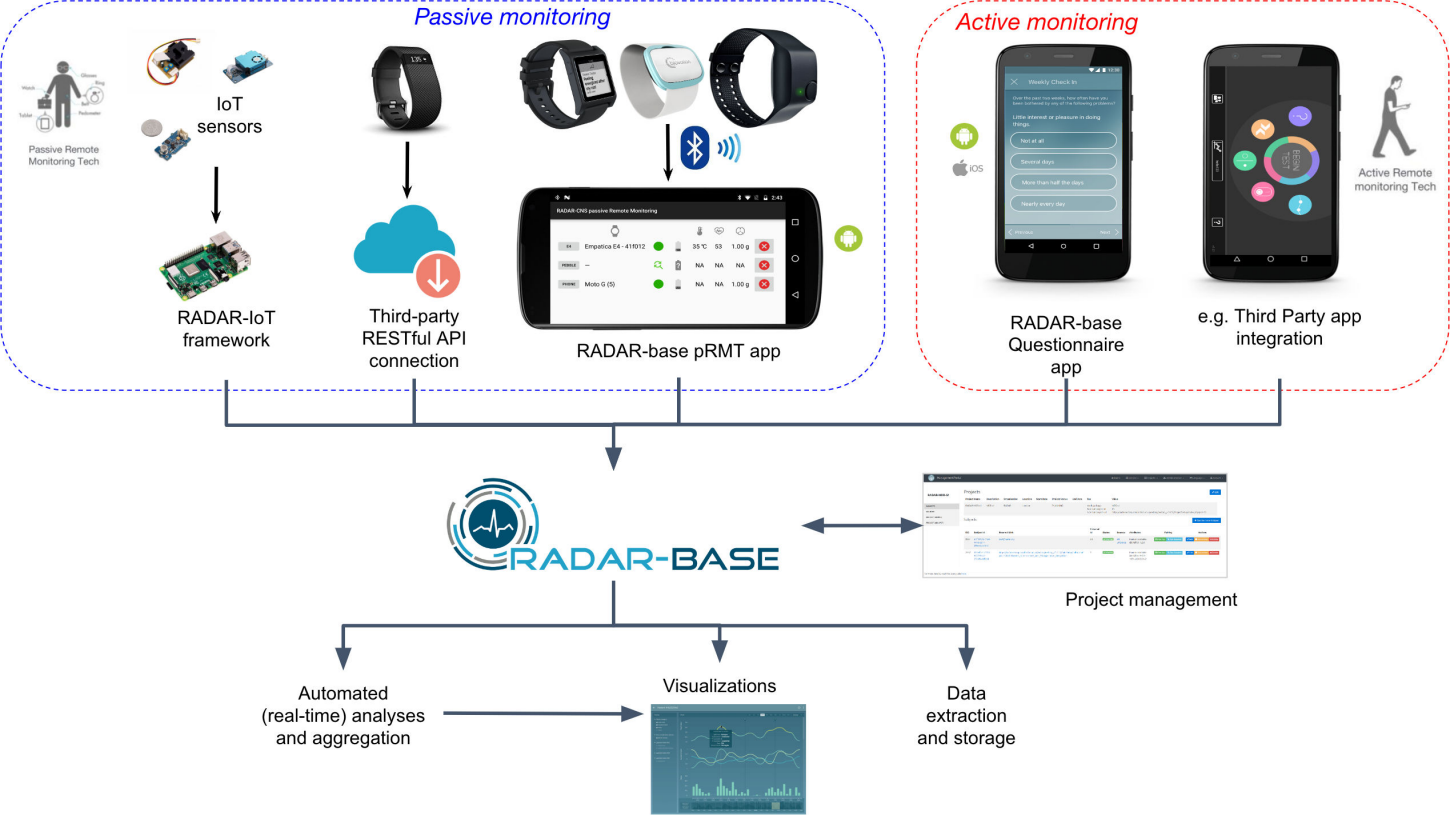
Overview of the RADAR-base platform. Current data sources: Empatica E4, Pebble 2, Fitbit, Biovotion, Faros, Garmin, Active Remote Monitoring questionnaire app and Passive Remote Monitoring app. API: application programming interface; IoT: Internet of Things.

High-quality longitudinal data collected for long periods and at scale are a key requirement for digital biomarker development. The widespread availability of smartphones, more capacious mobile networks, and the development of new wearable sensors have enabled measurement of a growing set of physiological and phenomenological parameters relevant to physical and mental diseases. To facilitate wearable and smartphone data remote collection at scale and digital biomarker development, the RADAR-base platform was developed and released under the open-source Apache 2 License in January 2018 [2,3]. RADAR-base comprises an Apache Kafka-based back end deployed onto Kubernetes infrastructure and 2 mobile apps. The cross-platform (Android, iOS) Cordova Active Remote Monitoring (aRMT) app for active monitoring of participants requires conscious action (eg, questionnaires, audio questions, timed tests), and the Passive Remote Monitoring (pRMT) app, a native Android app, does passive monitoring (without direct action from participants) via phone, wearable devices, and Internet of Things. A high-level overview of the platform is shown in Figure 1.

The aRMT app renders questionnaires using JavaScript Object Notation (JSON) definition files, which store data in key-value pairs. Within a single questionnaire file exists a collection of questions, each containing attributes such as field name, label or text, input type (checkbox, free text, etc), choices, and more. Subsequently, the aRMT app uses these files to display the questionnaire within the user interface. This facilitates the dynamic deployment of questionnaires for wide-ranging project requirements. Example questionnaires used in the aRMT app in existing projects include measures of self-esteem (Rosenberg Self-Esteem Scale), depression (8-item Patient Health Questionnaire), and ecological momentary assessment. These questionnaires include traditional question sets, while others take a different approach. For instance, the speech questionnaire requires users to record themselves reading a specific text or answering a question, rather than responding to written prompts.

The passive application runs in the background, requiring minimal or no input from participants. Data are collected from smartphone “sensors” and from integrated wearable devices. The catalogue of devices currently integrated into the pRMT app includes onboard Android smartphone sensors, Empatica E4, Pebble 2 smartwatch, Biovotion Everion, Faros 180 and 360, Fitbit, Garmin Vivosmart, and Oura Ring. Pluggable capability is provided to integrate new wearable devices offering a native software development kit (SDK) (eg, Empatica E4) or through third-party vendor’s Representational State Transfer Application Programming Interface (REST API) (eg, Fitbit via the back end REST Collector + REST-Authorizer for OAuth-2 Flows).

A common task is the exploration of collected raw data. RADAR-base includes capability for data aggregation, management of studies, and real-time visualization in Grafana dashboards [4]. In addition to the near real-time visualization provided by the dashboard, the RADAR-base platform includes a Python package designed for data processing, feature generation, and visualization. This package offers a range of standard tools for exploratory visualizations of collected data. It also simplifies the implementation of feature generation pipelines, allowing users to take data exported from a RADAR-base project and generate processed data (high-level features), along with any associated labels, in a format suitable for use with commonly used machine learning libraries.

### Objectives of This Study

The platform is used in a wide range of research and clinical studies and is available under a range of service models, depending on the requirement of the project. This paper extends the foundation laid by the initial iteration of the RADAR-base platform [3], describing its architecture and technical components in thorough detail. This work aims to encapsulate and outline a pertinent assortment of research and clinical studies that leverage the RADAR-base platform for data collection and digital phenotyping. The focus of this paper will be on distilling prevalent usage patterns and addressing the challenges encountered in using the platform for diverse research endeavors.

### Related Work and Comparison With Other Platforms

Numerous studies are validating digital biomarkers for disorders and their effectiveness, and studies [5] and [6] were conducted to assess the usefulness of digital biomarkers for mood and depression. Both studies had smaller cohorts of only 60 and 59 participants, respectively, which limits the studies’ findings. Digital biomarkers are being studied with a view to replacing or augmenting traditional markers for disorders and a number of barriers have been identified; these barriers include standardization and regulation, and studies are undergoing to address challenges and streamline digital biomarkers in health care [7]. Open source software platform for end-to-end digital biomarker development digital biomarker discovery pipeline was developed to standardize digital biomarker development. Digital biomarker discovery pipeline modules calculate and use resting heart rate, glycemic variability, insulin sensitivity status, exercise response, inflammation, heart rate variability, activity, sleep, and circadian patterns to predict health outcomes [8]. “mCerebrum: A Mobile Sensing Software Platform for Development and Validation of Digital Biomarkers and Interventions” supports high-rate data collections from multiple sensors with real-time assessment of data quality and development of digital biomarkers [9]. Guidelines for developing digital biomarkers are proposed in the study [10].

Intel Context Sensing SDK is a library for Android and Windows with specific context states; it, however, provides only front-end components [11]. The EmotionSense app is developed by the University of Cambridge to sense emotions with implications for psychological therapy and improving well-being; however, it is focused only on depression [12]. Medopad, now known as Huma, provides solutions for different health care issues with symptom tracking; this is a commercial solution and mainly focuses on phone sensors and active-monitoring methods [13]. Personal health INTERVENTION tool kit allows users to build health apps based on existing infrastructure [14]. ResearchKit is an open-source framework for building apps specifically for iOS. ResearchKit makes it easier to enroll participants and conduct studies; however, new wearable device integration requires strong programing skills and it does not include a data management solution [15].

ResearchStack is an SDK and user experience framework for building research study apps on Android, with a similar application domain as ResearchKit [16]. Both ResearchKit and ResearchStack provide software libraries, frameworks, and development tools that require extensive programing skills to create apps. A framework to create observational medical studies for mobile devices without extensive programing skills was presented [17]. LAMP (Learn Assess Manage and Prevent) platform [18] provides an app and back-end infrastructure for clinical relevant studies; app can adapt to different studies with input from patients.

One of the distinctive features of the RADAR-base platform is its use of Confluent platform technologies [19], which are built around Apache Kafka. This choice forms the foundation for a highly scalable end-to-end solution for event-driven messaging, capable of addressing diverse use cases such as high throughput, low-latency messaging, real-time data processing, and fault tolerance. Deployable as microservices with Kubernetes cluster [20], the platform offers seamless integration for new sensors and data sources with minimal effort. Number of new sensors are added to the platform since its inception for clinical studies. Minimal effort to integrate new sensors and devices offers opportunity to capture signals of various diseases and use cases.

There is a need for a systematic approach to assess the quality and use of digital biomarkers to ensure an appropriate balance between their safety and effectiveness. Coravos et al [21] outline key considerations for the development and evaluation of digital biomarkers, examining their role in clinical research and routine patient care. RADAR-base provides a secure and effective digital biomarker ecosystem, ensuring transparency of the algorithms, interoperable components with open interfaces to accelerate the development of new multicomponent systems, and high-integrity measurement systems.

## Methods

### Digital Phenotyping of Disease

**Figure 2.**
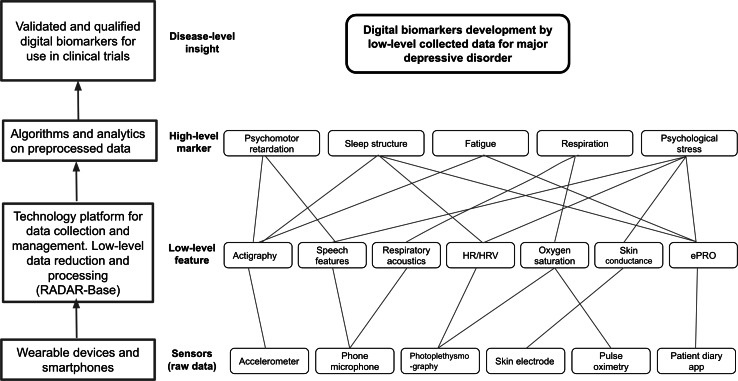
Collected raw data transition into high-level features which gives insight into major depressive disorder. ePRO: electronic patient-reported outcomes; HR: heart rate; HRV: heart rate variation.

A key feature of RADAR-base is its extensibility allowing new wearables and sensors to be readily integrated into the platform to collect new modes of data depending on the study requirements. Collected raw data from phone and wearable sensors can then be aggregated and converted into low-level features and subsequently high-level features, representing digital biomarkers. Figure 2 exemplifies the process for major depressive disorder (MDD) for wearable and phone sensor–collected data, for example, phone microphone–collected audio data can provide different speech features and respiratory acoustics that could help identify respiration and physiological stress; similarly, low-level acceleration provided–actigraphy features can be used to identify psychomotor retardation. Developed digital biomarkers may provide insight into the disorder and could be used in clinical trials to ascertain their usefulness in management of the disease.

The next two sections discuss the development of high-level features for MDD and epilepsy and how these features are being used to explore and manage disease. Textbox 1 shows selected features extracted from sensor data and Table 1 shows digital biomarkers developed for different disorder areas [22-24]. An open-source feature generation pipeline has been created to enhance and standardize the analysis of data generated by RADAR-base. This pipeline facilitates the extraction of features and biomarkers, enabling cross-disease symptom analysis. With its capabilities to ingest, analyze, visualize, and export RADAR-base data, the pipeline simplifies and establishes a convention for data scientists. This streamlines the process of feature-based analysis, ensuring consistency and enabling researchers to gain valuable insights from the data. Pipelines are readily extended and published on the RADAR-base pipeline catalogue [25].

Textbox 1. **Features extracted from the sensors integrated into the RADAR-base platform.**

**Apps**

- Active Remote Monitoring
- Passive Remote Monitoring

**Devices**

- Empatica E4
- Biovotion Everion
- Bittium Faros 180
- Faros 360
- Fitbit
- Garmin
- Smartphone
- Oura Ring

**Sensors/raw data**

- Acceleration
- Blood volume pulse
- Electrodermal activity
- Interbeat interval
- Temperature
- Blood pulse wave
- Galvanic skin response
- Heart rate
- Oxygen saturation
- Led current
- Photoplethysmogram raw
- Electrocardiogram
- Gyroscope
- Light
- Magnetic field
- Location
- Microphone
- Step count
- Usage event
- User interaction
- Activity levels
- Activity log record
- Intraday calories
- Intraday steps
- Resting heart rate
- Sleep classic
- Sleep stage
- SMS text message unread
- Bluetooth devices
- Phone battery level
- Phone contact list
- Garmin stress tracking
- Garmin relaxation
- Breathing timer
- Garmin Vo2max
- Garmin body
- Battery energy
- Monitor
- SpO2

**Selected features**

- Sleep duration
- Sleep architechture
- Sleep stability
- Sleep quality
- Sleep efficiency
- Sleep Fragmentation Index
- Sleep onset latency
- Sleep onset latency variance
- Sleep midpoint
- Sleep midpoint variance
- Insomnia
- Hypersomnia
- Unlock times/duration
- Unlock duration min/max
- Median interval between two unlocks
- Step epoch
- Daily step sum
- Moderate walking duration
- Maximum nonstop duration
- Maximum nonstop step count
- Activity level
- Actigraphy
- Respiratory acoustics
- Heart rate
- Heart rate variation
- Oxygen saturation
- Skin conductance
- Electronic patient-reported outcome
- Ambient light
- Activity
- Phone use
- Bluetooth
- Max/min/mean/SD of nearby Bluetooth devices (NBDC)
- NBDC entropy
- NBDC frequency features
- Location
- Location variance
- Moving time
- Moving distance
- Number of location clusters
- Location entropy
- Homestay
- Location frequency features
- Gait
- Median gait cycles
- Frequency of gait
- Median force
- Change in total sleep
- Social jet lag

**Table 1.** Digital biomarkers generated from extracted features and associated studies.

Digital biomarker	Device	Sensor/raw data	RADAR-CNS^a^	RADAR-AD^b^	AIMS-2-TRIALS^c^	ART^d^	BigData@Heart	RALPMH^e^	COVID-Collab
Total sleep	Fitbit/Garmin	Sleep stage	✓	✓	✓	✓	✓	✓	✓
Social interactions	Smartphone	Bluetooth	✓		✓	✓			
Gait patterns	Smartphone	Acceleration	✓	✓		✓			
Respiration	Garmin	Garmin respiration rate						✓	✓
Psychological stress	Garmin	Garmin stress			✓		✓	✓	✓
Phone use	Smartphone	User interaction	✓		✓	✓	✓		
Ambulatory mobility	Smartphone	Location	✓			✓			✓
Fatigue	Garmin	Garmin body battery energy						✓	✓
Seizures	Empatica E4/Biovotion Everion	Acceleration, EDA^f^, PPG^g^	✓						
Step	Fitbit/Garmin	Step count	✓	✓		✓			✓
Activity	Fitbit/Garmin	Activity logs	✓	✓	✓	✓		✓	✓
Speech	Smartphone	Microphone	✓		✓			✓	
Resting HR^h^	Fitbit/Garmin	PPG	✓	✓		✓		✓	✓
HR variability	Fitbit/Garmin	PPG	✓	✓		✓		✓	✓
Sleep variance	Fitbit/Garmin	Sleep stage	✓	✓		✓		✓	✓
Mobility variance	Smartphone	Location	✓	✓		✓		✓	✓
Respiratory acoustics	Smartphone	Microphone	✓	✓				✓	

a RADAR-CNS: Remote Assessment of Disease and Relapse—Central Nervous System.

b RADAR-AD: Remote Assessment of Disease and Relapse–Alzheimer Disease.

c AIMS-2-TRIALS: Autism Innovative Medicine Studies–2–Trials.

d ART: ADHD Remote Technology.

e RALPMH: Remote Assessment of Lung Disease and Impact on Physical and Mental Health.

f EDA: electrodermal activity.

g PPG: photoplethysmography.

h HR: heart rate.

#### Major Depressive Disorder

MDD is associated with a wide range of negative outcomes including premature mortality, reduced quality of life, and loss of occupational function, and it is often experienced alongside physical comorbidity and approximately 55% will go on to develop chronic depression, characterized by periods of recovery and relapse [26].

The pRMT app provides a comprehensive solution for activity monitoring by using wearable device sensors and smartphones to collect data without requiring any input from the wearer. It leverages a range of sensors, including GPS, accelerometer, gyroscope, communication logs, ambient noise and light levels, and screen interactions. Through this approach, the app can effectively and passively gather diverse data streams, enabling a seamless and unintrusive data collection process for various applications. These sensors along with the Fitbit watch have the potential to identify changes in sleep, communication, and activity patterns associated with depressive episodes.

**Figure 3.**
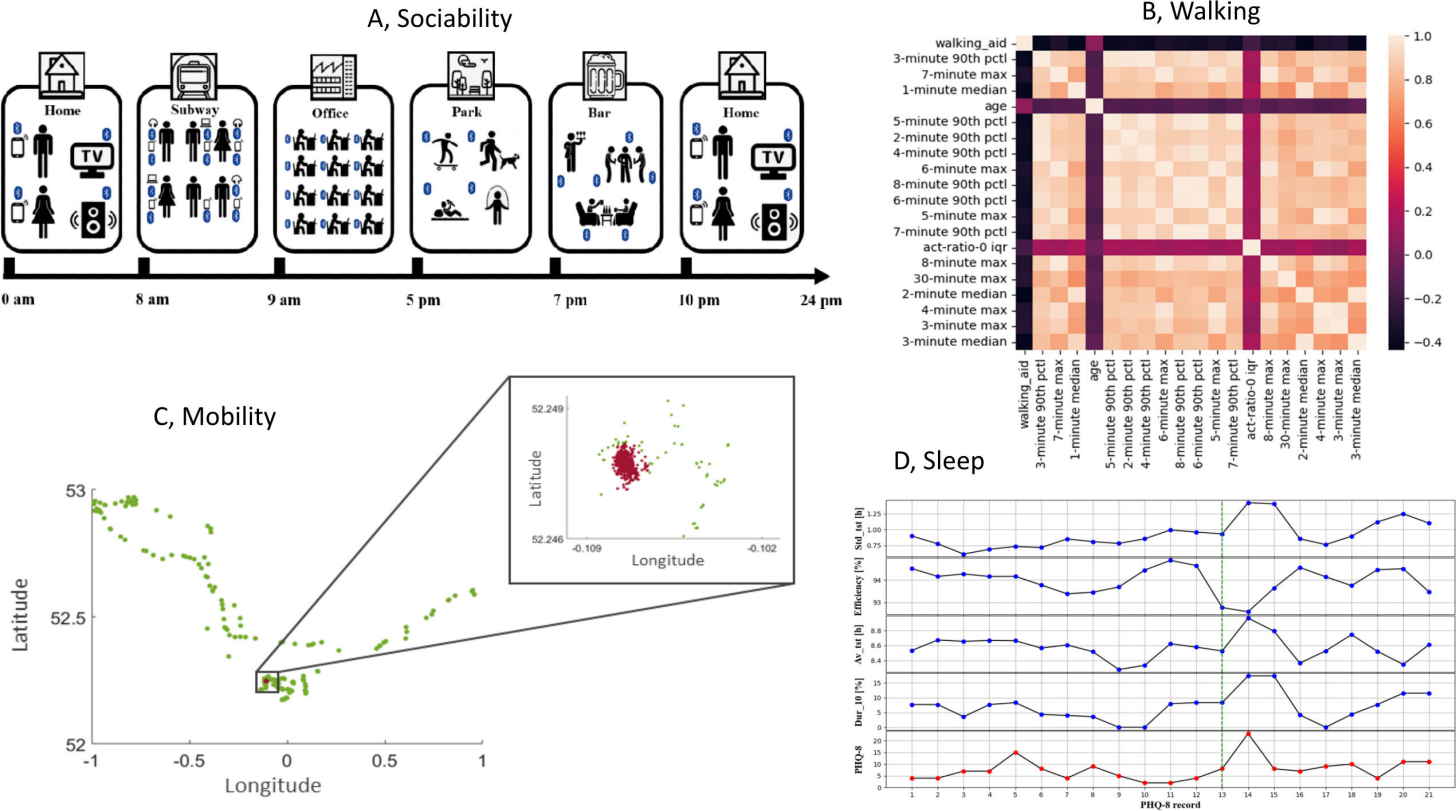
(**A**) A schematic diagram showing an individual’s nearby Bluetooth devices count (NBDC) in different scenarios in daily activities and life. (**B**) Pearson correlation heat map for top 20 features of walking data (median rankings from all models). (**C**) Exemplar geolocation data correspond to a biweekly segment of a study participant, The red dots denote an individual’s home location cluster, whereas longitude and latitude along the axes are expressed in decimal degrees. (**D**) The PHQ-8 scores and a select 4 sleep features of 1 participant with an obvious increasing trend in PHQ-8 score at 13th PHQ-8 record. PHQ-8: 8-item Patient Health Questionnaire.

The smartphone-embedded Bluetooth sensor can be used to record individuals’ local proximity information, such as the nearby Bluetooth device count (NBDC) that includes the Bluetooth signal of other phone users. The NBDC data have the potential to reflect changes in people’s behaviors and statuses during the depressive state [27]. An illustration is given in Figure 3.

Speech characteristics, such as speaking rate, pitch, pause duration, and energy, collected via smartphone microphones, have been used to detect depression with a prediction accuracy of 81.3% [28]. The aRMT app delivers validated questionnaires, cognitive games, speech tasks, or electronic diaries using the experienced sampling methodology to provide a fine-grained understanding of mood changes and stressors in the context of daily life. The aRMT app has been used to measure effect, cognition, and mood and behavior in real time, with evidence highlighting the increased validity of this methodology in comparison with traditional retrospective reports. The aRMT assessments of positive and negative affect have also been found to be reliably indicative of mood state and have been associated with MDD symptoms [29].

#### Epilepsy

**Figure 4.**
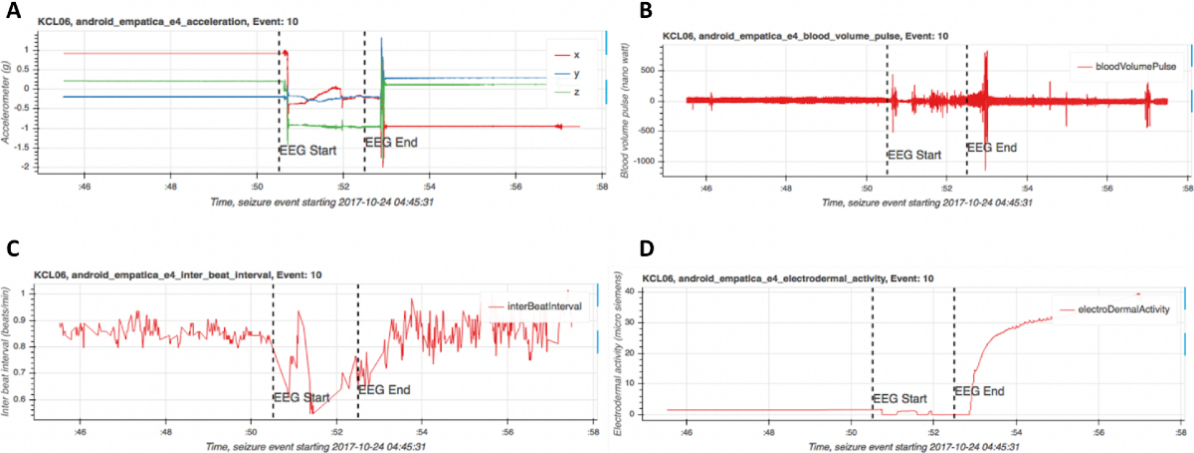
Empatica E4 sensor data. The area between the vertical dashed lines indicates a focal seizure with a motor component. (**A**) accelerometer, (**B**) photoplethysmography (PPG) blood volume pulse, (**C**) PPG interbeat interval, and (**D**) electrodermal activity. EEG: electroencephalogram.

Numerous epilepsy research studies based on epilepsy monitoring units have shown the possibility of capturing characteristic movement associated with myoclonic seizure manifestations using wearable sensors [30]. Using pRMT app–integrated wearable sensors, it is possible to record several signals associated with seizure including motor components, using inertial sensors such as accelerometry and surface electromyography, various features of heart rate variations captured by wearable electrocardiogram and photoplethysmography, and alteration of the autonomic nervous system with electrocardiogram, photoplethysmography, and electrodermal activity sensors, with different levels of signal and seizure detection accuracy. Figure 4 shows seizures detected with the collected data using the Empatica E4 wearable.

### Participant Recruitment Process

Participants are recruited using different methods, including through clinical services, hospitals, and remotely including through a citizen science approach, depending on the study requirements. A number of recruitment strategies are supported by the platform including the following:

1. All participants are recruited at once, and the study starts simultaneously.
2. Participants enter the study in a “batch” mode.
3. Participants are recruited continuously until the desired sample size or date is reached (“stream mode”).

Simultaneous recruitment from multiple sites is possible supporting recruitment of diverse population groups for the same study.

### Projects Using RADAR-Base Platform

Table 2 presents a summary of selected projects using the platform with disorders they are focused on, along with the cohort size and sensors being used. It also lists the main objectives of the project. A brief summary of the projects listed in Table 2 are provided in the next sections. In some projects, wearables data collected through the platform augment existing collected data, for example, historical clinical records or baseline assessments.

**Table 2.** Projects summary with disease area and study size including devices used and main objectives.

Project and disease area		Size n	Enrolled, n	Devices/data types	Main objectives
**RADAR-CNS** ^a^					
	Depression	600	623	Fitbit, phone sensors, questionnaires	Depressive relapse
	Epilepsy	200	145	Biovotion Everion, Empatica E4, questionnaires	Epilepsy seizure and preictal seizure detection
	Multiple sclerosis	500	430	Fitbit, Bittium Faros, phone sensors, questionnaires	Trajectory of disease, characterization, relapsing/remitting of disease symptoms
**ART-CARMA^b^**					
	Cardiometabolic risk factors	300	200 (ongoing)	Empatica EmbracePlus, phone sensors, questionnaires	Pretreatment initiation through to treatment initiation, titration, and the subsequent period
**ART^c^**					
	Attention-deficit/hyperactivity disorder	40	40	Fitbit, phone sensors, questionnaires	To establish a remote assessment and monitoring system for adults and adolescents with ADHD^d^
**RADAR-AD^e^**					
	Alzheimer disease	200	229	Fitbit, phone sensors, questionnaires	Feasibility of remote-monitoring technologies for AD^f^
**AIMS-2-TRIALS^g^**					
	Autism	500	300 (ongoing)	Empatica E4, Fitbit, phone sensors, questionnaires	Biology of autism to tailor treatments and develop new therapies and medicines
**BigData@Heart**					
	Atrial fibrillation	160	160	Phone sensors, questionnaires	Comparison of 2 strategies of rate control, based either on initial treatment with digoxin or β-blockers
**DynaMORE^h^**					
	Mental health	—^i^	—	Phone sensors, questionnaires	Developing an in-silico model of stress resilience
**CONVALESCENCE**					
	Long-term effects of COVID-19	800	363 (ongoing)	Garmin Vivoactive, phone sensors, questionnaires	Characterization, determinants, mechanisms, and consequences of the long-term effects of COVID-19
**COVID-Collab**					
	COVID-19	15,000	17,667	Fitbit, questionnaires	Behavior and physical and mental health occur in response to COVID-19 infection, persistent symptoms, and the pandemic in general
**RALPMH^j^**					
	Lung disease	60	60	Garmin Vivoactive, phone sensors, Questionnaires	Feasibility of remote-monitoring technologies for high-burden pulmonary disorders
**EDIFY**					
	Eating disorder	500	10 (ongoing)	Oura Ring, phone sensors, questionnaires	Delineating illness and recovery trajectories to inform personalized prevention and early intervention in young people
**UNFOLD**					
	Psychosis	50	—	Questionnaires	To characterize the processes involved in developing an identity as a person in recovery
**Jovens na Pandemia & MAAY Study**					
	Depression	280	—	Phone sensors, questionnaires	Remotely monitor behavioral and symptom changes associated with behavioral interventions in children and adolescents

a RADAR-CNS: Remote Assessment of Disease and Relapse—Central Nervous System.

b Attention-Deficit/Hyperactivity Disorder Remote Technology Study of Cardiometabolic Risk Factors and Medication Adherence.

c ART: ADHD Remote Technology.

d ADHD: attention-deficit/hyperactivity disorder

e RADAR-AD: Remote Assessment of Disease and Relapse–Alzheimer Disease.

f AD: Alzheimer disease.

g AIMS-2-TRIALS: Autism Innovative Medicine Studies–2–Trials.

h DynaMORE: Dynamic Modelling of Resilience.

i Not available.

j RALPMH: Remote Assessment of Lung Disease and Impact on Physical and Mental Health.

#### Remote Assessment of Disease and Relapse–Central Nervous System

Remote Assessment of Disease and Relapse–Central Nervous System (RADAR-CNS) was a cohort study that developed new ways of monitoring MDD, epilepsy, and multiple sclerosis using wearable devices and smartphone technology. Patients’ data were collected continuously for 24 months [31]. More than 1200 participants took part in the study in different disease areas, and participant recruitment was done via clinics and hospitals. Different study protocols with different wearable devices were used for each disease. Participants were recruited from 6 different sites from different countries.

Digital biomarkers developed through the remotely collected data give a better understanding of the diseases and will help clinicians manage them timely [27,32,33].

#### Attention-Deficit/Hyperactivity Disorder Remote Technology Study of Cardiometabolic Risk Factors and Medication Adherence

The Attention-Deficit/Hyperactivity Disorder Remote Technology Study of Cardiometabolic Risk Factors and Medication Adherence (ART-CARMA) aims to obtain real-world data from the patient’s daily life to explore the extent to which attention-deficit/hyperactivity disorder (ADHD) medication and physical activity, individually and jointly, may influence cardiometabolic risks in adults with ADHD. The second objective is to obtain valuable real-world data on adherence to pharmacological treatment and its predictors and correlates. The long-term goal is to use collected data to improve the management of cardiometabolic disease in adults with ADHD and to improve ADHD medication treatment adherence and the personalization of treatment [34]. For this cohort, 2 study sites in London and Barcelona are concurrently recruiting the participants using the platform.

#### ADHD Remote Technology

The ADHD Remote Technology (ART) was a pilot project focused on developing a novel remote assessment system for ADHD. ART assessed the feasibility and validity of remote researcher-led administration and self-administration of modified versions of 2 cognitive tasks sensitive to ADHD, a 4-choice reaction time task (Fast task) and a combined Continuous Performance Test/Go No-Go task (CPT/GNG) [35]. A cohort of 40 participants was recruited, 20 controls and 20 patients with ADHD.

#### Remote Assessment of Disease and Relapse–Alzheimer’s Disease

Remote Assessment of Disease and Relapse–Alzheimer’s Disease (RADAR-AD) aimed to transform Alzheimer disease patient care through remote assessment using mobile technologies such as smartphones or fitness trackers [36]. The project developed the technology to identify which clinical or physiological features, digital biomarkers, can be measured remotely to predict deterioration in function. RADAR-AD created a pipeline for developing, testing, and implementing remote measurement technologies with patients involved at each stage. Complete details of the study protocol and pipeline development are explained in the study by Muurling et al [37]. It was an augmentation study in which 300 participants took part. Three different categories of participants were recruited: controls, mild cognitively impaired or prodromal Alzheimer disease, and Alzheimer dementia.

#### Autism Innovative Medicine Studies–2–Trials

The Autism Innovative Medicine Studies–2–Trials (AIMS-2-TRIALS) program includes a range of studies to explore how autism develops, from before birth to adulthood, and how this varies in different people. AIMS-2-TRIALS is looking for biological markers which indicate whether a person has or may develop particular characteristics [38]. AIMS-2-TRIALS collects both active and passive data in clinical assessment settings and in home-based and ambulatory settings. Fitbit is used for remote data collection, and Empatica E4 is used for local data collection at hospitals. Digital markers could help identify those who may ultimately benefit from particular treatments. Medicines will also be tested to help with social difficulties, repetitive behaviors, and sensory processing. Remote-monitoring data are augmenting the clinical data.

#### Rate Control Therapy Evaluation in Permanent Atrial Fibrillation (BigData@Heart)

The Rate Control Therapy Evaluation in Permanent Atrial Fibrillation (RATE-AF) study was designed to compare 2 strategies of rate control, based on either initial treatment with digoxin or β-blockers in 160 patients with atrial fibrillation (AF) in need for rate control therapy. Monitoring with wearable devices, phone sensors, and questionnaires was conducted over a continuous 6-month period. Objectives of the project included discovering new phenotypes, developing reliable subphenotyping, and informing new taxonomies of heart failure based on a better understanding of underlying disease processes. Sleep, heath rate, heart rate variation, and activity data were collected to develop new phenotypes. This work is additionally supported by the EU IMI2 BigData@Heart major program [39].

#### Dynamic Modelling of Resilience

Dynamic Modelling of Resilience (DynaMORE) generated the first personalized in-silico model of mental health in the face of adversity or stress resilience. The model is based on and validated against unique multiscale longitudinal real-world empirical data sets, collected through neuroimaging, experimental assessments, questionnaires, and remote monitoring using the pRMT app and a wearable device. The model will substantially deepen scientific understanding of the mechanisms of resilience, supporting the creation of mechanistically targeted interventions for the primary prevention of stress-related disorders. On this basis, DynaMORE developed an entirely new mobile health (mHealth) product incorporating the RADAR-base platform that will include model-based prognostic tools for real-time and real-life monitoring of at-risk subjects and for automated decision-making about timed, personalized interventions.

#### CONVALESCENCE

CONVALESCENCE is focused on the characterization, determinants, mechanisms, and consequences of the long-term effects of COVID-19, providing the evidence base for health care services [40]. It is an existing large longitudinal cohort being further characterized and augmented by incorporating wearables data. Deep phenotyping and remote assessment using mobile devices and smartphones through the RADAR-base platform is being used to identify subclinical damage or dysfunction in individuals with long-term COVID-19.

#### COVID-Collab

COVID-Collab is a citizen science project with members of the public volunteering to donate their wearable data and complete diagnosis and symptom questionnaires. The main aim was to investigate the ongoing COVID-19 outbreak: (1) establish whether wearable data can be used to diagnose COVID-19 infection and (2) characterize the disease symptoms and evolution. A key feature of the study is the use of wearable data to investigate changes in mental health and physiological measurements such as heart rate during infection with coronavirus [41].

#### Remote Assessment of Lung Disease and Impact on Physical and Mental Health

**Figure 5.**
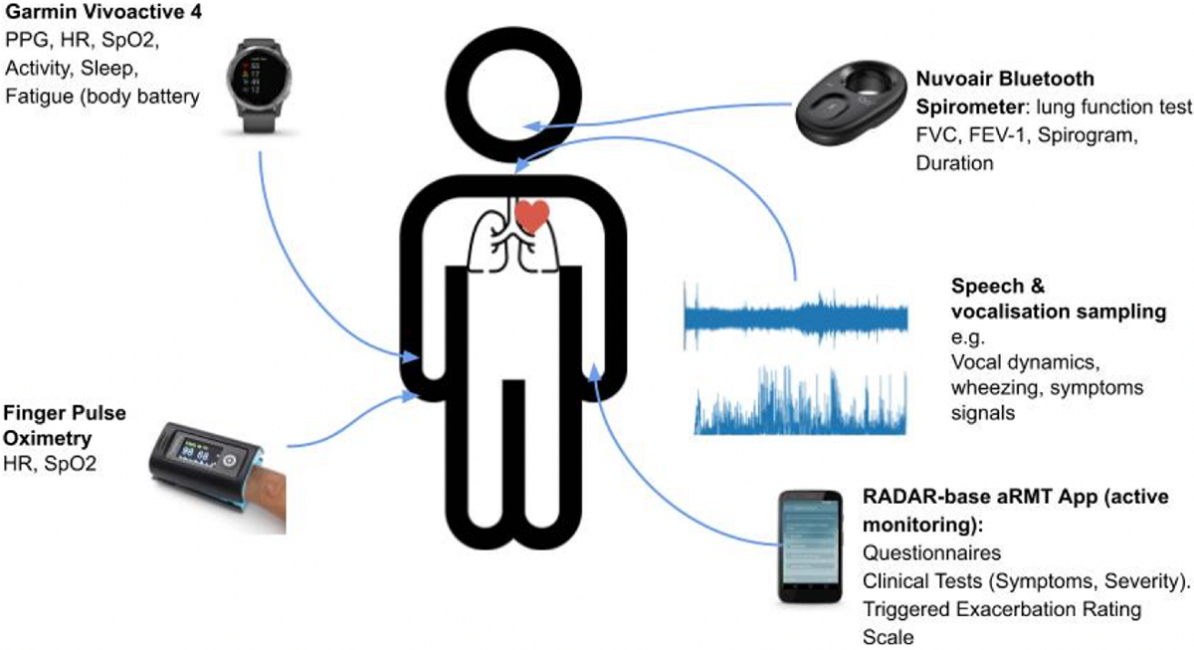
Various data sources will be used to collect both active and passive data to gain a unique perspective into patient health in the Remote Assessment of Lung Disease and Impact on Physical and Mental Health lung disorder study. aRMT: Active Remote Monitoring; FEV-1: forced expiratory volume in the first second of expiration; FVC: forced vital capacity; HR: heart rate; PPG: photoplethysmography.

Chronic lung disorders such as chronic obstructive pulmonary disease and idiopathic pulmonary fibrosis are characterized by exacerbations and decline over time. Twenty participants were recruited in each of 3 cohorts (chronic obstructive pulmonary disease, idiopathic pulmonary fibrosis, and posthospitalization COVID). Data collection is being done remotely using the RADAR-base platform for different devices, including Garmin wearable devices and smart spirometers, mobile app questionnaires, surveys, and finger pulse oximeters. The Remote Assessment of Lung Disease and Impact on Physical and Mental Health (RALPMH) project focuses on the feasibility of remote monitoring in chronic lung disorders and provides a reference infrastructure for future studies [42]. Figure 5 shows an overview of the RALPMH study.

#### Eating Disorder (EDIFY)

The objective of the EDIFY study is to undertake a longitudinal comparison of the biopsychosocial symptom profiles of those with early- and late-stage eating disorders (EDs) and recovery trajectories of those with early-stage EDs. This will provide evidence that will help inform decision-making of targeted intervention and preventative treatments across EDs for those with early and more progressed forms of illness. ED patients do not like to wear any wearable device or have any information displayed on their phone regarding their calories and daily workout. The Oura ring is being experimented for the first time with ED patients at scale as it provides no feedback to the patients during the study.

### Ethical Considerations

Prior to initiating each project, we obtained approval for each study from the relevant ethics committee and conducted a data protection impact assessment (DPIA), which is equivalent to an institutional review board in some countries. Each study adhered to its unique data collection protocol, and both the General Data Protection Regulation or DPIA and the protocol were approved by the research ethics committee and the data protection officer, respectively. In the study by Ranjan et al [3], comprehensive information regarding data protection, privacy, and the process of pseudonymization is expounded.

### Using RADAR-Base Platform and Hosting Models

RADAR-base is of interest to a wide range of mHealth communities, from academic research to industry and wearable vendors interested in collecting data remotely or integrating new data sources into the platform. The platform is freely available as an open-source (Apache 2 License) GitHub repository [43]. More details of the platform can be found on the official RADAR-base website [44]. A detailed quickstart, deployment details, and developer documentation are made available on the platform Confluence Wiki [45]. Docker images for all the components are available at Docker Hub [46] and a Kubernetes stack is also available for the deployment [47]. The aRMT app questionnaires and protocol implementation is explained in “RADAR-base/RADAR-aRMT-protocols” [48]. An exemplar DPIA that explains the data collection procedure is shared in Multimedia Appendix 1. In principle, 3 hosting models are available for using the platform.

#### Self-Hosting

The platform, along with its accompanying apps and related elements, is open source, allowing users to host it on private or local servers and tailor it to suit their study requirements. Under the self-hosting model, users possess full autonomy over deployment, infrastructure, and data collection.

#### Supported Hosting

Under the supported hosting model, a third-party provider of RADAR-base can deploy the platform on local or public cloud such as Amazon Web Services. The costing and support arrangements will be determined by factors such as the study’s duration, participant count, and data throughput, primarily based on sensor types selected. In this setup, users will share control over the infrastructure and data with the RADAR-base team. Integration with electronic case report forms such as REDCap (Research Electronic Data Capture) also allows more sensitive data to be segregated from the managed service and be retained entirely under the user’s control.

#### Fully Managed Hosting

This option is available to completely outsource the deployment, and hosting to a third party provides the platform deployed on their infrastructure and hosts projects as a service to, for example, researchers.

## Results

### Overview

**Figure 6.**
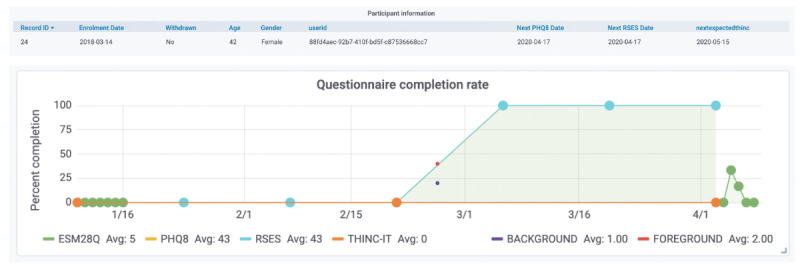
Active Remote Monitoring app questionnaire completion rate from a single patient from the Remote Assessment of Disease and Relapse—Central Nervous System major depression study. ESM: experienced sampling methodology; PHQ8: 8-item Patient Health Questionnaire; RSES: Rosenberg Self-Esteem Scale.

A large number of mental and physical health research studies have used the RADAR-base platform for remote data collection with funding from many major funding agencies. This includes more than 50 use cases exploring more than 30 disorder areas with more than 150,000 participants enrolled to date. Major disease areas that are using the platform are MDD, eating disorder, multiple sclerosis, ADHD, autism, epilepsy, atrial fibrillation, Alzheimer Disease, and COVID-19. Projects using the platform are collecting various health parameters depending on the disease area requirement. Data collected relate to cognition, mood, voice, digital usage, geolocation, and heart rate, to name a few. Figures6  7 show examples of the status of collected data, their compliance, and quality for different studies. Numerous challenges addressed by the platform include completeness of data, quality and accuracy of data, participant engagement, and remote data collection.

**Figure 7.**
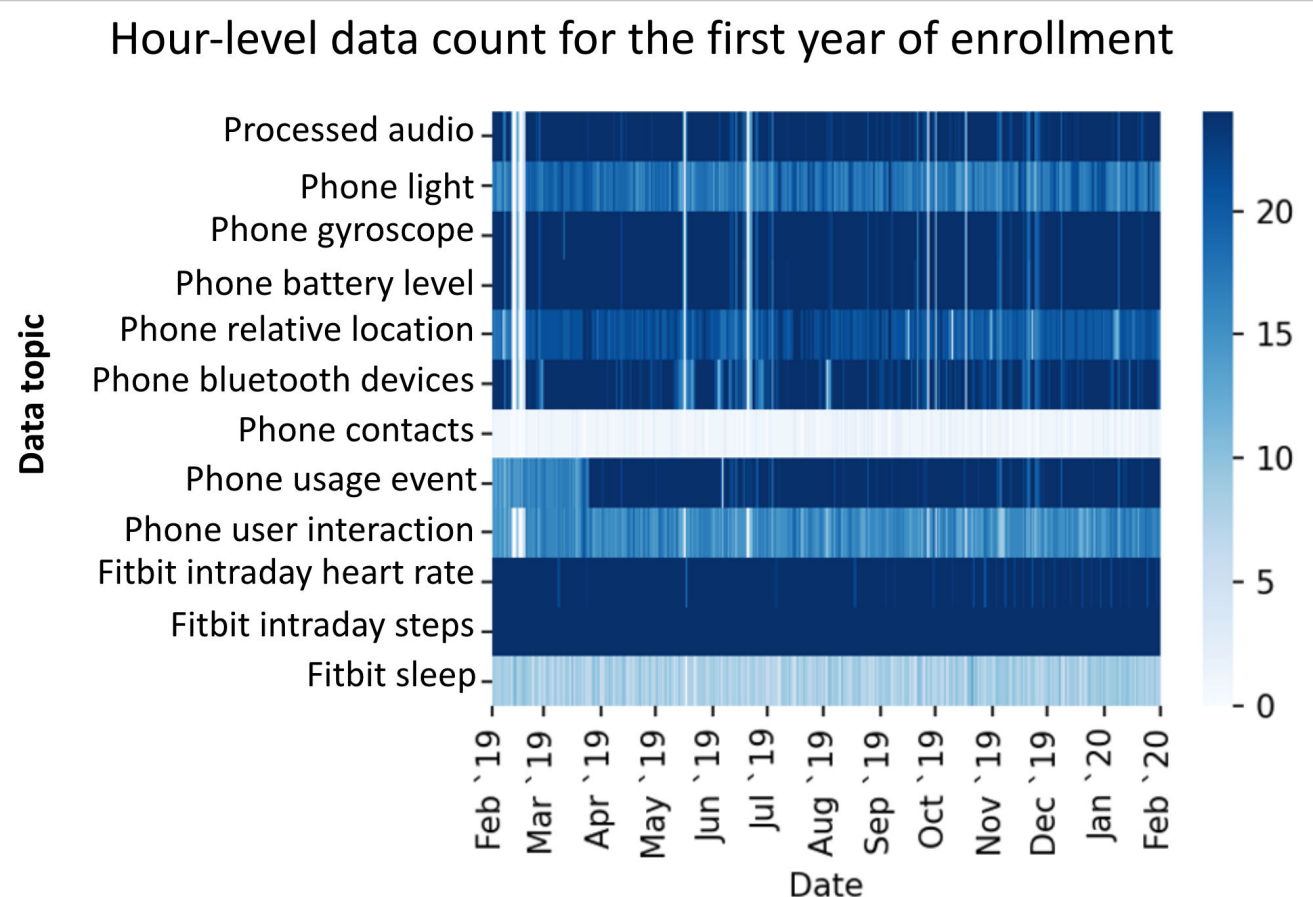
Contiguity of phone sensor data over the first year of enrollment collected through RADAR-base for a patient in the Remote Assessment of Disease and Relapse—Central Nervous System major depressive disorder study. The intensity of the color represents how many hours in a day a particular modality is present.

### Digital Biomarker Development

The RADAR-base platform has effectively transformed low-level sensor data into digital biomarkers through feature generation pipelines. This process involves extracting relevant characteristics and patterns from the raw data, enabling the creation of meaningful and actionable insights. These digital biomarkers hold immense potential in various disease areas, aiding clinicians in making informed decisions, facilitating early intervention, and contributing to the prevention of relapse.

### Participant Retention, Engagement, and Data Quality

Studies using the RADAR-base platform for data collection and feature extraction have reported insights into data quality, participant engagement, and retention [49,50]. In the Major Depressive Disorder Study, 623 participants were enrolled, with 79.8% (n=497) of them participating for the maximum study duration (11-24 months). In addition, further analysis revealed that wearable data stream had the highest data available rate (n=407 participants with more than 90% data completeness and n=99 participants with more than 50% completeness) across all data streams and found several indicators impacting participant engagement and retention, such as age and mental health status [50]. These findings illustrate the feasibility of remote data collection for clinical applications and provide the insights and experiences for future mobile health studies.

## Discussion

Results suggest that the RADAR-base platform can effectively gather critical health data outside of traditional clinical settings and generate digital biomarkers, improving continuous monitoring and management of physical and mental conditions. It provides a platform that focuses on safety and effectiveness by ensuring transparency in the algorithms used to generate biomarkers. In addition, the interoperable components with open interfaces in RADAR-base facilitate the development of new multicomponent systems. This interoperability ensures that various digital biomarker sources can be integrated into a comprehensive and cohesive platform for health care purposes. Moreover, RADAR-base emphasizes high-integrity measurement systems, which means that the data collected and the biomarkers generated are reliable and accurate. This emphasis on data quality is essential for building trust in the digital biomarker ecosystem and encouraging its adoption in clinical research and routine patient care.

Overall, the systematic approach and emphasis on safety, effectiveness, transparency, and interoperability offered by RADAR-base can contribute to the advancement of digital biomarkers and their integration into health care systems, ultimately benefiting patient outcomes and medical research.

## Supplementary material

10.2196/51259 Multimedia Appendix 1 Exemplar RADAR-base platform data protection impact assessment.

## Abbreviations

ADHD: attention-deficit/hyperactivity disorder
AF: atrial fibrillation
aRMT: Active Remote Monitoring
DPIA: data protection impact assessment
ED: eating disorder
JSON: JavaScript Object Notation
LAMP: Learn Assess Manage and Prevent
MDD: major depressive disorder
mHealth: mobile health
NBDC: nearby Bluetooth device count
pRMT: Passive Remote Monitoring
REDCap: Research Electronic Data Capture
REST: Representational State Transfer
SDK: software development kit
