# Supplementary material for: Digital Phenotyping of Mental and Physical Conditions: Remote Monitoring of Patients Through RADAR-Base Platform
Source: JMIR Ment Health. 2024 Oct 23;11:e51259. doi: 10.2196/51259 (PMC11524428; doi:10.2196/51259)
Supplement: Multimedia Appendix 1 [file mental-v11-e51259-s001.docx]

**Generalised DPIA for RADAR-base**

*Below is the template for the Data Protection Impact Assessment (DPIA) for utilising the RADAR-based platform. The DPIA addresses various questions related to data collection and processing, considering different scenarios where the platform handles data from various applications and devices. Additionally, RADAR-base's data storage and processing options are discussed.*

# **DATA PROTECTION IMPACT ASSESSMENT**

This document is designed to identify privacy and data protection risks. Please complete with as much detail as possible and return the document to the Information Compliance team for review.

**Screening Questions – Do I need a Data Protection Impact Assessment?**

**Answer according to the Project/Study**

| Question | Y/N |
| --- | --- |
| Are you going to be collecting information about vulnerable data subjects? (e.g., children, vulnerable adults, patients) | Y |
| Will the information be disclosed to organisations or people who have not previously had access to the information? (e.g. a research partner or institution, another department within King’s) | Y |
| Will the information be used in a way that it is not currently used? (e.g. to send text messages about revision sessions) | Y |
| Will you make decisions about individuals or act against them as a result of collecting or using the information? (e.g. to decide whether to offer an additional tutoring service based on exam results) | N |
| Are you using new technology that could be intrusive? (e.g. biometrics or facial recognition) | N |
| Is the information that you are collecting or using sensitive information (e.g. health records, criminal records, religious beliefs – see definitions in procedure for full list) or information that may be perceived as sensitive to individuals (e.g. salary, bank account details, copies of passports)? | N |
| Will you contact the individuals in a way they might find *intrusive* (e.g. telephone calls, emails to personal email addresses) | Y |
| Is the processing of personal data ‘large scale’?  Are many data subjects involved?  Does the project cover a large geographical area?  Does it cover a very long period?  Is there a variety of different types of data being processed? | N |
| Is more than one dataset containing personal data being matched or combined? | N |
| Will systematic monitoring of individuals be taking place? (e.g. CCTV) | Y |
| Is the processing otherwise likely to result in a high risk to the freedoms or rights of individuals?  Will the processing prevent data subjects from exercising their rights? See guidance on [data subject rights](https://internal.kcl.ac.uk/about/secretariat/business-assurance/compliance/gdpr/GDPR-Guide-to-Subject-Rights.pdf) and [Guidelines on Data Protection Impact Assessment (DPIA) and determining whether processing is “likely to result in a high risk” for the purposes of the Regulation 2016/679](https://ico.org.uk/media/about-the-ico/events-and-webinars/2172578/a29wp-dpia-guidance-201710.pdf) | N |
| Will personal data be transferred out of the EU/EEA? | N |

If your answer to any of these questions is ‘yes’ a full assessment will usually be required. If not, but personal data is still being processed, please contact Information Compliance with a summary of your project for further advice.

**Step 1: Why do I need a Privacy Impact Assessment?**

**Answer according to the Project/Study**

| Project Summary/Description of Processing |
| --- |
| *Please provide a summary of your project. You can attach a brief proposal or other document if available. Please state if more than one processing activity is covered by this DPIA. Please include the nature, scope, purposes and context of the processing and describe any relevant hardware, software, networks or people who may be relevant. Please also include reference to any relevant codes of practice, legislation or funding contracts which may be relevant. What are the projected outcomes of the project?*  To establish a remote assessment and monitoring system for adults and adolescents with ADHD that can be applied to both research and clinical long-term monitoring of symptoms, impairments and health-related behaviours, with the ultimate aim to support self - management and clinician decision-making, and improve outcomes.  Remote measurement technology (RMT) offers unprecedented opportunities for addressing current challenges in ADHD research and treatment. Adult ADHD is associated with an increased risk for detrimental outcomes, including educational and occupational failure, and criminal behaviour, yet individual long-term outcomes are highly variable, with some individuals remitting in young adulthood, appearing free of clinical impairment. A better understanding of predictors and markers of different longterm ADHD outcomes is required for developing interventions that aim to halt adverse developmental trajectories. A remote ADHD assessment and monitoring battery would enable detailed, frequent, long-term, real-world data collection on the clinical symptoms of ADHD and co-occurring disorders, functional and cognitive impairments, and health behaviours (such as exercise and sleep) on large sample sizes. RMT would offer a shared tool for more detailed data collection and larger collaborations than have previously been possible. This application reflects the essential first phase in this process: we will develop and pilot the new remote measures for ADHD.  We will perform a pilot study with 20 adolescents and adults with ADHD, to establish the feasibility of the remote assessments and monitoring of each participant over a 10-week period, to establish the validity of the remote versions of cognitive tasks in comparison to lab-based assessments, and to obtain pilot data on potential digital biomarkers for ADHD. We will also obtain data from a control sample of 20 adolescents and adults without ADHD. Participants with ADHD will also be asked to identify an informant (a parent, partner or a close friend) who could complete informant-report versions of the active monitoring questionnaires on ADHD symptoms and impairment, and irritability.  The aim is to develop and pilot a novel remote assessment system for adolescents and adults with ADHD that incorporates active and passive monitoring using mobile and web technologies.  Outcomes for this pilot study are:   - Feedback from participants on the acceptability of the measures and data on adherence to RMT - Concurrent validity of remote versions of cognitive tasks (CPT/GNG and fast task): how well data obtained using self-administration at home correlates with data obtained in the lab (self-administration in the presence of a research worker). Also to confirm case-control differences on the cognitive tasks. - Within-person fluctuation in each remote monitoring measure over the 10-week period, to establish how sensitively we can capture changes in symptoms and impairments over this period - Association of ADHD symptoms and their fluctuation with the other remote monitoring measures and their fluctuation - Association of baseline lab measures with the remote monitoring measures and their fluctuation |
| Screening Questions |
| *Please provide more details about the ‘Yes’ responses to the screening questions*  **Answer Depending on the Project/Study**  ART is an observational non-randomised, non-interventional study, using commercially available wearable technology and smartphone sensors, representing no change to the usual care or treatments of participants due to participation.  Participants with ADHD will attend a baseline lab session with a research worker at the SGDP Centre, Institute of Psychiatry, Psychology and Neuroscience, where they will: (1) undergo an ADHD diagnostic interview to confirm ADHD diagnosis, (2) undergo an IQ and working memory assessment, (3) perform two cognitive tasks of CPT/GNG and the fast task (while research worker present), (4) receive a training session on the use of wearable devices, mobile sensors and Active RMT (aRMT) smartphone app, and (5) complete the web-based Redcap baseline questionnaires. This session will take approx. 3 h. Control participants will be assessed on the same measures, except that instead of the full ADHD diagnostic interview, they will complete the ADHD symptom and impairment questionnaire on the smartphone.  Passive monitoring, using the smartphone and activity monitor, starts from this initial session and will be ongoing for 10 weeks for each participant. Passive monitoring means that the data are collected automatically using the devices without any active input from the participant. Participants need to wear the wrist-worn activity monitor for the 10 weeks and to carry the smartphone with them throughout this period.  Active monitoring, which involves the participant completing clinical symptom questionnaires on the smartphone and the two cognitive tasks on their home PC or laptop will take place 3 times during the pilot study: baseline remote assessment 1 week after the baseline lab assessment, 1^st^ follow-up remote assessment 4 weeks after the baseline remote assessment and 2^nd^ follow-up remote assessment 8 weeks after the baseline remote assessment. Each participant is therefore in the study for 10 weeks. While participants are completing the cognitive tasks, we will ask participants to record themselves. This monitoring will help ensure that participants are attending the tasks. Participants will use a PC/laptop webcam to record themselves and data collected will be automatically saved to a Dropbox folder that only the participant and immediate research team can access. Recordings will later be reviewed by the research team to ensure that the participant was attending throughout the cognitive tasks.  During the baseline lab session, the participants with ADHD will also be asked to identify an informant (a parent, partner or a close friend) who could complete informant-report versions of the active monitoring questionnaires on ADHD symptoms and impairment, and irritability, using the web-based Redcap. The research worker will contact the informant and invite him/her to complete the questionnaires at each of the 3 remote active monitoring time points. |
| Example of Dataset |
| *Please provide an example of your dataset, including all the categories of personal data that will be processed.*  <https://github.com/RADAR-base/RADAR-Schemas/tree/master/specifications>  Fitbit data will use dummy data (dummy name and email address) in the account information (maybe with the exception of the participant height).  REDCap Questionnaires and forms:   - ADHD diagnostic interview (DIVA) - IQ and working memory (WASI-II, WAIS-IV) - Cognitive tasks (CPT/GNG, Fast Task) - ‘About you’ questionnaire (socio-demographic, medical history, service use, experience with technology) - Autism symptoms (AQ) - ADHD symptoms and impairment (BAARS-IV, BFIS) - Anxiety and depression questionnaires (PHQ-8, GAD-7) - Reactive-Proactive Aggression Questionnaire (RPQ) - Affective Reactivity Index (ARI-s, self-report) - Informant-report questionnaires (ADHD symptoms and impairment: BAARS-IV, BFIS; Affective Reactivity Index: ARI)   Personal data: name, date of birth, telephone number, email address, home address, diagnosis.  Informants name, telephone number and email address. All personally identifiable information is kept in REDCap, which is only accessible by the immediate research team. |

**Step 2: The Data Flow**

| Collection of Data | |
| --- | --- |
| What data will be collected and from whom? How many individuals?  **Answer Depending on the Project/Study** | 20 participants with ADHD, 20 informants of the ADHD participants, and 20 control participants. Data will be collected from the baseline lab and through wearables, smartphone and personal pc/laptop.  Baseline lab assessments   - ADHD diagnostic interview (DIVA) - IQ and working memory (WASI-II, WAIS-IV) - Cognitive tasks (CPT/GNG, Fast Task) - REDCap (web-based) self-report questionnaires (‘About you’: socio-demographic, medical history, service use, experience with technology; Autism: AQ)   Remote active data collection:   - Cognitive tasks (CPT/GNG, Fast Task) - ADHD symptoms and impairment (BAARS-IV, BFIS) - Anxiety and depression questionnaires (PHQ-8, GAD-7) - Reactive-Proactive Aggression Questionnaire (RPQ) - Affective Reactivity Index (ARI-s, self-report) - REDCap (web-based) informant-report questionnaires (ADHD symptoms and impairment: BAARS-IV, BFIS; Affective Reactivity Index: ARI)   Passive data collection:   - Fitbit wristband activity measure: physical activity and sleep - Smartphone sensors: relative location, weather, light, social communication and interaction, overall smartphone use - For participants who use social networking sites (Twitter, Facebook) social networking use will be collected   eCRF Forms   - Contact details (removed from data analysis and only used for study management team) |
| Why is this data collection necessary?  **Answer Depending on the Project/Study** | The purpose of this study is to develop and pilot a novel remote assessment system for attention-deficit/hyperactivity disorder (ADHD) that incorporates active and passive monitoring using mobile and web technologies. The long-term vision for this project is to establish a remote assessment and monitoring system for adults and adolescents with ADHD that can be applied to both research and clinical long-term monitoring of symptoms, impairments and health-related behaviours, with the ultimate aim to support self-management and clinician decision-making, and improve outcomes. |
| Who will be collecting the data?  **Answer Depending on the Project/Study** | The research team will collect data from the baseline lab assessments (see above) and RADAR-base and REDCap (specialised and standardised data collection platforms) will collect the remote data. |
| What method of collection will you use?  **RADAR-base platform related question** | Data will be collected with a combination of our mobile apps, and the https://radar-base.org platform. |
| Will you be obtaining consent? If so, how will this be captured and documented?  **Answer Depending on the Project/Study** | Yes, the consent forms and information sheets will be emailed to the participants for them to read prior to their baseline lab assessment. When they arrive for the baseline lab assessment, they will go through the information sheet with a research worker, allowing opportunity for questions to be asked, after which they have the opportunity to sign the consent form. |
| What information will be provided to the data subjects about how their personal data is handled? *Please attach any privacy notices you intend to use*  **Answer Depending on the Project/Study** | Information sheet and consent forms will provide details about how participants personal data is handled. Participants will also have the opportunity before giving sign consent to ask the research worker any questions regarding this. All the personal information will be handled according to GDPR rules and this will be explained in detail to the participants. |
| What is the [lawful basis](https://ico.org.uk/for-organisations/guide-to-the-general-data-protection-regulation-gdpr/lawful-basis-for-processing/) for processing the personal data? E.g. necessary for a contract, necessary for our public task, consent, etc  **Answer Depending on the Project/Study** | The lawful basis and condition for processing personal data is necessary for the performance of this task which is carried out in the public interest or in the exercise of official authority vested in the controller (Article 6(1)e) and processing is necessary for scientific research purposes in accordance with Article 89(1) based on Union or Member State law which shall be proportionate to the aim pursued, respect the essence of the right to data protection and provide for suitable and specific measures to safeguard the fundamental rights and the interests of the data subject (Article 9(2)j).  <https://eugdpr.org/> |
| How many individual datasets will you collect?  **Answer Depending on the Project/Study** | 60 (20 participants with ADHD, 20 informants of the ADHD participants, and 20 control participants) |
| Use of Data | |
| What decisions/actions will be taken using the data?  **Answer Depending on the Project/Study** | This pilot study will provide essential data needed for subsequent larger-scale studies that will monitor individuals with ADHD for longer periods of time and for studies that will compare individuals with ADHD to individuals with other psychiatric disorders. |
| Who will have access to the data?  **Answer according on the Project/Study** | Data will be accessible only to members of the immediate research team, following GDPR guidelines. |
| Will the data be transferred to third parties? Is so, are any of these third parties outside of the EEA? Are there any data sharing agreements, or contracts with data processors in place? Please see the [guide to data sharing.](https://internal.kcl.ac.uk/about/secretariat/business-assurance/compliance/gdpr/GDPR-Sharing-Personal-Data.pdf) Will a third party be making decisions about the data?  **RADAR-base platform related question** | **In case of Fitbit data**  Participant ID will be pseudonymised and data that will be identifiable will be de-identified. Data will be securely stored on servers managed by the Institute of Psychiatry, Psychology and Neuroscience, King’s College London, and also on Fitbit 3^rd^ party servers, which can provide additional security and backup. These data will only be linked back to participant personal information by specific members of the research team.  Fitbit will collect and store data on Fitbit servers on participants' physical activity and sleep. Data processed by Fitbit will be pseudonymised (dummy information, such as dummy name and email address, will be entered into the Fitbit accounts). Google Firebase will also store some event log data generated by the app.  Participants will be given information about 3rd parties accessing portions of pseudonymised data and be directed towards Fitbit’s own privacy policy <https://www.fitbit.com/uk/legal/privacy-policy>  No data access outside EEA. Fitbit will collect pseudonymised data in accordance to their privacy policy (<https://www.fitbit.com/uk/legal/privacy-policy>), as described above. Third parties will not be making decisions about the data.  **In case of Empatica Embrace**  Participants’ personal data will be pseudonymised. Data will be securely stored on servers managed by the Institute of Psychiatry, Psychology and Neuroscience, King’s College London, and also on Empatica 3rd party servers, which are GDPR compliant and are present in the EEA. Empatica will collect and store data on Empatica servers on participants’ pulse rate, pulse rate variability, temperature, Sp02, electrodermal activity and physical activity. Data processed by Empatica will be pseudonymised (dummy information, such as dummy name and email address, will be entered into the Empatica accounts). Google Firebase will also store some event log data generated by the app. Participants will be given information about 3rd parties accessing portions of pseudonymised data and be directed towards Empatica’s own privacy policy. No data access outside EEA. Third parties will not be making decisions about the data. <https://www.empatica.com/privacy/>  **In case of Oura Ring and Embrace Plus**  Same as the case of Fitbit mentioned above.  **Note:** No research data will be shared with above mentioned device vendors for research and analysis purposes, unless otherwise explicitly agreed. |
| How will any third parties process the data?  **RADAR-base platform related question** | **In case of Fitbit data**  Fitbit will process data according to their privacy policy <https://www.fitbit.com/uk/legal/privacy-policy>. The data processed by Fitbit will be pseudonymised (dummy information, such as dummy name and email address, will be entered into the Fitbit accounts).  **In case of other devices Google Cloud and Amazon AWS or Microsoft Azure**  Google Cloud is used to receive data from participant’s phones and store it until it is extracted to KCL-based servers. Google is not permitted to use the data in any other way or for their own purposes. Amazon AWS may be used in addition or in replacement of KCL servers for the Fitbit data collection work. Fitbit data is already consented by the contributing participants who are using their own devices, they will only be donating this data to the study. Both Google Cloud and Amazon AWS are GDPR compliant.  **Note:** No third party will process the research data for research and analysis purposes until unless explicitly mentioned and agreed. |
| Who will be responsible for accuracy and corrections, or considering deletion of personal data if requested in line with the rights of the data subjects?  **Answer according to the Project/Study** | A data manager within the project will be assigned the tasks for this purpose. |
| How will you handle any objections to processing or, where relevant, withdrawal of consent?  **Answer according to the Project/Study** | Participants can voluntarily withdraw from the study at any time, but any data collected up until this point may still be used. A research worker within the project will be assigned the tasks for this purpose. |
| Will data portability be possible?  **Answer according to the Project/Study** | No. |
| Storage, Access (including third party providers) | |
| Where will the data be stored?  **RADAR-base platform related question** | **Scenario 1- Local Storage**  All electronic data is stored on a specific SFTP server and the Rosalind server (KCL’s private network, <http://rosalind.kcl.ac.uk/>), information is transmitted in a secure encrypted way. This follows similar procedures previously established for the RADAR-CNS project. An exception is the Fitbit data which first go to the Fitbit server before coming back to the KCL servers (but no other  data are held by Fitbit).  **Scenario 2- Third Party Storage**  Data will be stored on Amazon S3 storage/ Google Storage/ Microsoft Azure which are GDPR compliant. |
| How will access be restricted?  **RADAR-base platform related question** | **Scenario 1- Local Storage**  Authorised access to secure Storage Server using RSA encryption over SSH, as mentioned above (SFTP and Rosalind). Access Control Lists (ACLs) and user-groups used to control object level permissions in the storage for each user.    **Scenario 2- Third Party Storage**  P.I will decide data access and authorisation for research data which is securely stored on GDPR compliant third-party clouds/storage. |
| Will data be anonymised? *Please see the* [*ICO code of practice on anonymisation*](https://ico.org.uk/media/1061/anonymisation-code.pdf)  **Answer according on the Project/Study** | Yes. |
| Who is the data controller (who determines the manner and purpose of the processing)?  **Answer according on the Project/Study** | King’s College London. |
| Retention and Deletion | |
| Estimated length of data cycle  **Answer according on the Project/Study** | 12 months (data collection phase to last 6 months) |
| For how long will you keep the data? Please include justification  **Answer according on the Project/Study** | The data will be retained in their original form for 10 years, after which only de-identified data are stored. |
| How will it be destroyed?  **Answer according on the Project/Study** | All personally identifiable information will be permanently deleted. Chief Investigator Prof Jonna Kuntsi will be responsible for requesting deletion, a README file on the folder with instructions about the intent to delete after expiry (10 years) will be held. |
| Security | |
| What measures will be in place to keep the data safe and secure? | Data will be placed with the secure network of King’s College and RSA asymmetric public key encryption will be applied to access. KCL IT has been consulted on this. |
| Will the data be transferred outside the EU/EEA? If so under what [grounds](https://ico.org.uk/for-organisations/guide-to-the-general-data-protection-regulation-gdpr/international-transfers/)?  **Answer according on the Project/Study** | No. We will use the Dropbox EU servers only, to ensure data is only stored within the EU/EEA. The Dropbox EU servers are GDPR compliant. |
| Will children’s data (generally under 13 years old) be collected as part of the project? If ‘yes’, how will consent be obtained from a parent/guardian?  **Answer according on the Project/Study** | No. |
| Will the data of other vulnerable individuals be processed? If so what measures will be in place to protect them? | All the data will be stored pseudonymised and highly restricted access will be given to specific authorised researchers. Industry grade security will be used to secure data access.  <https://www.fitbit.com/uk/legal/privacy-policy>  <https://www.dropbox.com/en_GB/security>  <http://rosalind.kcl.ac.uk/> |

**Step 3: Consultation**

Should any other persons/departments be consulted about this project? Please list all relevant stakeholders who will be affected by this processing.

*Please enter the comments of any persons here*

No other people/departments apart from the project investigators.

Have any data subjects been consulted for their views on this processing?

*Please enter the comments of any persons here*

Andrea Bilbow – service user representative, President of ADHD Europe and an external partner in the project – has been involved in the planning of the study from the beginning and will continue to be involved throughout the project. We will also form an advisory group, involving individuals with ADHD, to provide input. In addition, study participants (individuals with ADHD and controls) will be asked to provide feedback on the measures and procedures after this pilot study is completed.

Has an Equality Analysis been completed for this project?

*King’s has a responsibility to consider the impact of decisions on individuals beyond how we process personal data. We have an obligation to demonstrate our considerations for protected groups. Please enter the summary from a completed Equality Analysis or explain why it is not relevant to the processing.*

**Step 4: Identifying the data protection risks and mitigating actions**

| Data protection issues/risks identified  *See Appendix 2 for examples and consult the Information Compliance team* | Recommended actions to mitigate the risks  *See Appendix 3 for examples and consult the Information Compliance team* | Residual risks  *Are the initial risks eliminated, reduced or the same? If reduced, what risk remains?* | Date actions implemented/ to be implemented |
| --- | --- | --- | --- |
|  |  |  |  |
|  |  |  |  |

**Step 5: DPO advice**

The DPO has reviewed this DPIA and advises that the processing can proceed as outlined, subject to the above actions being implemented.

*DPO signature, date and comments*

**OR**

The DPO has reviewed this DPIA and advises that the processing should not proceed as outlined due to the high residual risks. Processing cannot proceed without consulting the ICO first.

*DPO signature, date and comments*
